# Impact of device size and thickness of Al_2_O_3_ film on the Cu pillar and resistive switching characteristics for 3D cross-point memory application

**DOI:** 10.1186/1556-276X-9-692

**Published:** 2014-12-23

**Authors:** Rajeswar Panja, Sourav Roy, Debanjan Jana, Siddheswar Maikap

**Affiliations:** Thin Film Nano Tech. Lab., Department of Electronic Engineering, Chang Gung University, 259 Wen-Hwa 1st Rd., Kwei-Shan, Tao-Yuan 333 Taiwan

**Keywords:** Resistive switching, Al_2_O_3_, Cu pillar, 3D memory, CBRAM

## Abstract

Impact of the device size and thickness of Al_2_O_3_ film on the Cu pillars and resistive switching memory characteristics of the Al/Cu/Al_2_O_3_/TiN structures have been investigated for the first time. The memory device size and thickness of Al_2_O_3_ of 18 nm are observed by transmission electron microscope image. The 20-nm-thick Al_2_O_3_ films have been used for the Cu pillar formation (i.e., stronger Cu filaments) in the Al/Cu/Al_2_O_3_/TiN structures, which can be used for three-dimensional (3D) cross-point architecture as reported previously Nanoscale Res. Lett.9:366, 2014. Fifty randomly picked devices with sizes ranging from 8 × 8 to 0.4 × 0.4 μm^2^ have been measured. The 8-μm devices show 100% yield of Cu pillars, whereas only 74% successful is observed for the 0.4-μm devices, because smaller size devices have higher Joule heating effect and larger size devices show long read endurance of 10^5^ cycles at a high read voltage of -1.5 V. On the other hand, the resistive switching memory characteristics of the 0.4-μm devices with a 2-nm-thick Al_2_O_3_ film show superior as compared to those of both the larger device sizes and thicker (10 nm) Al_2_O_3_ film, owing to higher Cu diffusion rate for the larger size and thicker Al_2_O_3_ film. In consequence, higher device-to-device uniformity of 88% and lower average RESET current of approximately 328 μA are observed for the 0.4-μm devices with a 2-nm-thick Al_2_O_3_ film. Data retention capability of our memory device of >48 h makes it a promising one for future nanoscale nonvolatile application. This conductive bridging resistive random access memory (CBRAM) device is forming free at a current compliance (CC) of 30 μA (even at a lowest CC of 0.1 μA) and operation voltage of ±3 V at a high resistance ratio of >10^4^.

## Background

It is known that commercial FLASH memory approaches its scaling limit in sub-20-nm technology node. As it is an alternative solution to replace the FLASH, resistive random access memory (RRAM) becomes a center of attraction to the researchers because of its simple metal-insulator-metal (MIM) structure with low voltage operation (<3 V), high speed operation, and high scalability potential (<10 nm) [1–5]. Although many switching materials have been reported for the RRAM applications, the Al_2_O_3_ as a switching material has been reported a few [6–9]. The amorphous Al_2_O_3_ film has energy gap of 6.2 to 8.8 eV [10, 11], dielectric constant of 9 [12], and Gibbs free energy of -1,582.3 kJ/mole at 300 K [13], which can help to have good resistive switching properties. Wu et al. [6] have described a TiN/Al_2_O_3_/Pt RRAM device with a current compliance (CC) of sub-20 μA and high operating voltage of +8/-4 V. Wu et al*.* [14] have reported the Al/Al_2_O_3_/Pt RRAM devices with an unstable RESET current of 1 μA. Lin et al*.* [7] have reported the resistive switching characteristics using a Ti/Al_2_O_3_/Pt structure with a high CC of 10 mA and operating voltage of ±3 V. We have reported the resistive switching memory characteristics using an IrO_*x*_/Al_2_O_3_/IrO_*x*_-NDs/Al_2_O_3_/W structure with a CC/voltage of 500 μA/±4 V previously [8]. The resistive switching phenomena using an IrO_*x*_/AlO_*x*_/W structure with a CC of 200 μA in cross-point architecture have been also reported by us [9]. In this regard, many chalcogenide materials in the conductive bridging resistive random access memory (CBRAM) devices have also been reported by many groups [15–23]. Basically, silver (Ag) or Cu is used as an oxidizing electrode, and the metal ions are migrated through the chalcogenide material under a positive bias on the Ag or Cu electrode. Recently, the AlO_*x*_ material in the CBRAM devices using the Ag (or Cu)/Al_2_O_3_/bottom electrode (BE) structures [24–26] has emerged great interest to the researchers due to its high compatibility with the fabrication of conventional CMOS devices. Goux et al. [24] have reported the CBRAM devices using a Cu-Te/Al_2_O_3_/Si structure at a CC of 5 μA and operating voltage of +0.5/-1 V. In this case, they have used a thin layer (approximately 3 nm) of Al_2_O_3_ film as an active material and device size was large 1 × 1 mm^2^. Sleiman et al. [25] have investigated the Cu/AlO_*x*_/W CBRAM devices with a CC of 100 μA and operating voltage of ±2 V. The thickness of AlO_*x*_ layer is 20 nm, and a large device size is 1 × 1 mm^2^. Belmonte et al. [26] have described the resistive switching characteristics in 90-nm Cu/TiW/Al_2_O_3_/W 1T1R integrated cell, and they have introduced the TiW as a buffer layer to protect Cu diffusion. In this case, the thickness of Al_2_O_3_ layer is 3 nm. The memory device could be operated at 25 μA with operation voltage of ±2 V. It is noticed that the Al_2_O_3_ material has been used in the CBRAM devices using bi-layers and different structures and thicknesses. However, impact of device size and thickness of a single Al_2_O_3_ layer using the Cu/Al_2_O_3_/TiN CBRAM devices have not yet been reported.

On the other hand, one of the main challenges of CBRAM is to achieve high device uniformity and integration density. To have high-density memory, conventional three-dimensional (3D) FLASH has been conducted through-silicon-via (TSV) [27–30], which acts as interconnect in each stack. But one of the bottlenecks of TSV is high cost and large integration area. Joblot et al. [31] have reported the capability of Cu pillar for 3D interconnection technology. So, the conventional TSV technique of 3D FLASH can be also replaced by Cu pillar formation (i.e., stronger Cu filament in CBRAM structure) through 3D cross-point memory architecture for future below 11-nm technology node, which has been demonstrated previously [32]. In this case, each stack of cross-points memory could be connected through Cu pillar which would be a promising way for achieving ultra-high-density memory application. Therefore, the device size-dependent Cu pillar characteristics in the Cu/Al_2_O_3_/TiN structures have been also investigated for potential application.

In this study, impact of the device size and thickness on the Cu pillars and resistive switching memory characteristics using a single Al_2_O_3_ layer in the Cu/Al_2_O_3_/TiN CBRAM devices has been investigated. The amorphous Al_2_O_3_ film with a thickness of 18 nm and the device sizes are observed by transmission electron microscope (TEM) image. By measuring 50 randomly picked devices, the empirical result tells that large size devices are promising for future Cu pillar applications. From cumulative probability of 50 devices, the Cu pillars can reach up to a high CC of 70 mA. Hence, the strong metallic path inside the Al_2_O_3_ layer can be used as a connecting way among the different stacks of high-density 3D memory devices. Long read endurance of 10^5^ cycles of the large size devices at a high read voltage of -1.5 V ensures about the robustness of the Cu pillars or metallic paths. In addition, thickness-dependent resistive switching memory characteristics are also observed. Superior CBRAM characteristics such as high switching yield of 88%, lower average RESET current of approximately 328 μA, and acceptable resistance ratio of 9.6 are obtained for the 0.4-μm devices with a 2-nm-thick Al_2_O_3_ film under low voltage of ±2 V, which is due to lower diffusion of Cu ions into the Al_2_O_3_ films under external bias. The resistive switching mechanism is based on the formation and dissolution of the Cu filaments into the Al_2_O_3_ films. Good data retention ability of >48 h is also obtained. This CBRAM device shows forming-free current-voltage (*I*-*V*) characteristics under a CC of 30 μA, even at lowest CC of 0.1 μA.

## Methods

A silicon-dioxide (SiO_2_) with a thickness of 200 nm was deposited on 8-in. Si wafer for device isolation. Then, a titanium-nitride (TiN) layer as a BE was deposited on SiO_2_/Si substrate. The thickness of TiN BE was approximately 200 nm. A SiO_2_ film with a thickness of 150 nm was deposited for via-hole design. The via-holes with different sizes of 0.4 × 0.4 to 8 × 8 μm^2^ were patterned. To do the lift-off process, the pattern was coated by photo-resist (PR). Then, the via-hole and top electrode (TE) regions were opened. The Al_2_O_3_ layers with different thicknesses ranging from 2 to 20 nm were deposited by rf sputtering. The Al_2_O_3_ target with purity of 99.9% was used. During the deposition, argon (Ar) flow rate was 25 sccm. The deposition power and pressure were 80 W and 30 mTorr, respectively. The Cu as a TE was deposited by thermal evaporation. The thickness of Cu was approximately 40 nm. The deposition rate was 0.5 Å/s. To avoid oxidation of Cu at elevated temperature, aluminum (Al) as a capping layer was deposited by using the same thermal evaporator. The thickness of Al was approximately 160 nm. Deposition rate was 1 Å/s. Finally, lift-off process was performed to get the Al/Cu/Al_2_O_3_/TiN CBRAM devices. The memory device size and microstructure of an Al_2_O_3_ film was investigated by using TEM-JEOL 2100F system with energy of 200 keV and resolution of 0.2 nm. During electrical investigations of Cu pillar and resistive switching, the bias was applied on the Cu TE while the BE was grounded. The 20-nm-thick Al_2_O_3_ film was used to investigate the Cu pillars because the stronger Cu filament will be formed under external positive bias on the TE. Other thicknesses of 2 to 10 nm were used to study the resistive switching characteristics. Fifty devices for each size and thickness of Al_2_O_3_ were measured randomly. All the electrical measurements were performed by Agilent 4156C precision semiconductor parameter analyzer (Agilent Technologies, Inc., Santa Clara, CA, USA).

## Results and discussion

**Figure 1:**
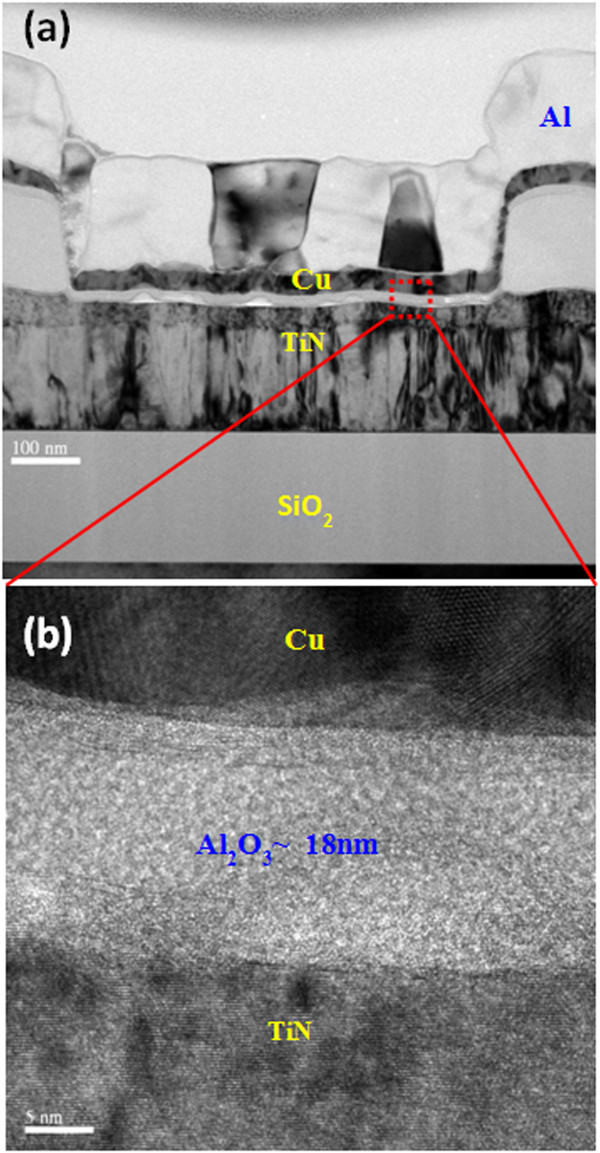
**TEM images of an Al/Cu/Al**_**2**_ **O**_**3**_ **/TiN structure. (a)** TEM image shows the device size of 0.5 × 0.5 μm^2^. **(b)** HRTEM image shows Cu/Al_2_O_3_/TiN structure. The thickness of insulating layer is approximately 18 nm.

Figure 1a shows TEM image of an Al/Cu/Al_2_O_3_/TiN via-hole device. All layers are observed clearly. The device size of approximately 0.5 × 0.5 μm^2^ is observed. Figure 1b shows high-resolution TEM image inside the via-hole region. The thickness of Al_2_O_3_ layer is approximately 18 nm, including a thin TiO_*x*_N_*y*_ layer on the TiN surface. This Al_2_O_3_ film shows amorphous. Due to the thicker Al_2_O_3_ film, the stronger Cu filament (or pillar) could be formed inside the via-hole region for 3D cross-point memory application, which has been discussed below.

**Figure 2:**
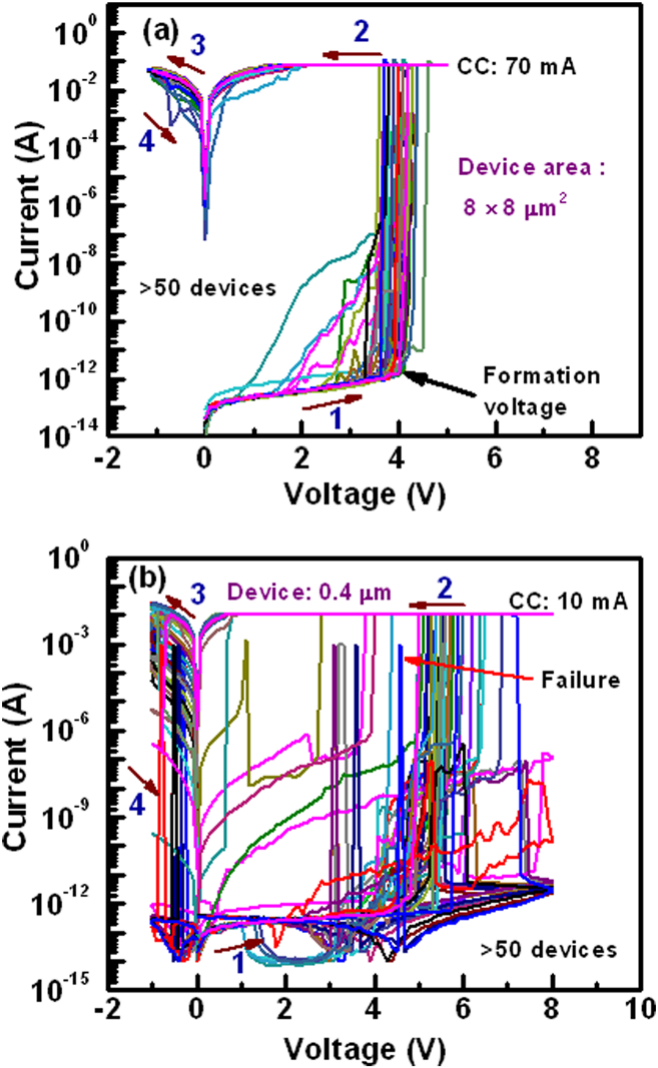
**Current-voltage characteristics of the Cu pillars.** *I*-*V* characteristics of arbitrarily measured 50 devices with device size of **(a)** 8 × 8 μm^2^ under a high CC of 70 mA and of **(b)** 0.4 × 0.4 μm^2^ under a CC of 10 mA. The smallest size devices have largest failure of the Cu pillars, due to the Joule heating. The thickness of the Al_2_O_3_ layer is 20 nm.

**Figure 3:**
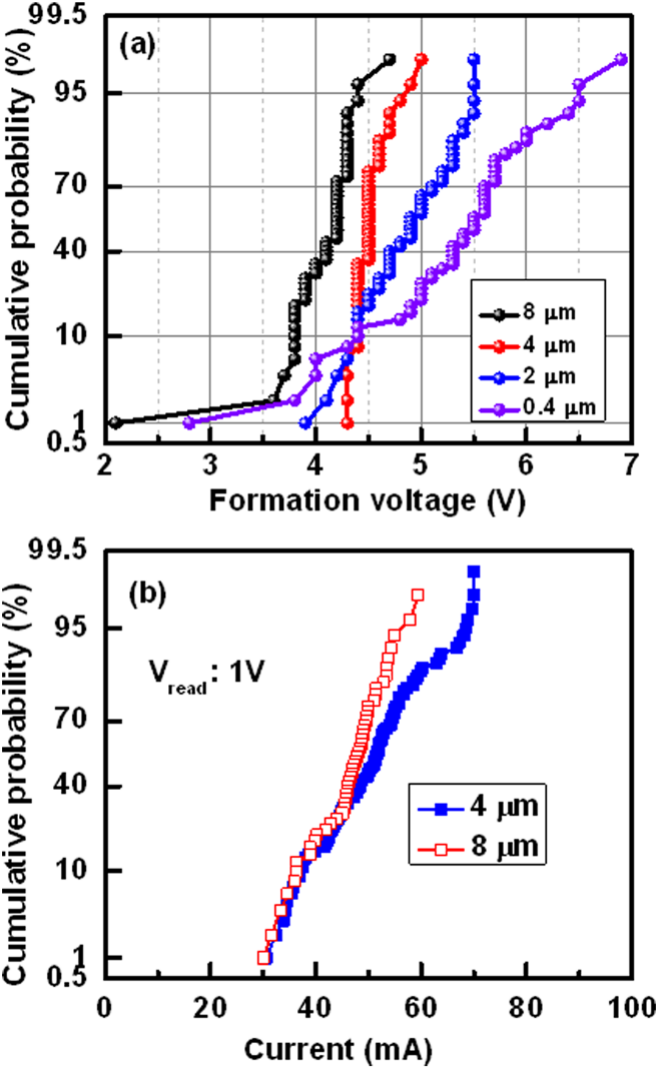
**Statistical distribution of formation voltage and current of the Cu pillars. (a)** Formation voltage increases with decreasing device area which suggests the higher Cu diffusion rate for large size devices. **(b)** Uniform current distribution at LRS of larger device sizes implies the probability of similar conduction path of the Cu pillars.

The *I*-*V* characteristics of randomly measured 50 pristine devices with two different sizes viz. 8 × 8 and 0.4 × 0.4 μm^2^ are shown in Figure 2. The thickness of Al_2_O_3_ film is 20 nm. The sweeping voltage direction is shown by the arrows 1 to 4, which also follows as 0 → +5 → 0 → -1.1 → 0 and 0 → +8 → 0 → -1 → 0 V for the devices with large and small sizes, respectively (Figure 2a,b). It is found that all 8-μm devices are operated at a high CC of 70 mA whereas many of the 0.4-μm devices show failure to reach even at a CC of 10 mA. By applying bias of -1 V on the TE, the 8-μm devices do not show RESET and few 0.4-μm devices show RESET. This suggests that the Joule heating burns the small size devices at a high current as well as device size-dependent filament diameter. Heat dissipation of larger size devices is higher than the smaller size devices. Thermal conductivities of Cu, Al, Al_2_O_3_, SiO_2_, TiN, and Si materials are 398, 244, 25.08, 1.38, 28.84, and 148 W/m/K, respectively [33]. This implies that heat will be dissipated through top electrode contact than the other sides. Therefore, the area of top electrode contact as well as device size will help to reduce heating effect, especially, when the device is operated at a high current of >10 mA. If the device does not show RESET, then stronger Cu filament (or pillar) is formed into the Al_2_O_3_ layer. The formation voltages (*V*_form_) for the 8-, 4-, 2-, and 0.4-μm devices at 50% probability are 4.2, 4.5, 4.9, and 5.5 V, respectively (Figure 3a). Therefore, the value of *V*_form_ increases with decreasing the device sizes owing to lower leakage current as well as lower defects into the Al_2_O_3_ layer. On the other hand, the formation energy is lower for larger size devices than the smaller one owing to the higher diffusion rate of Cu ions with the area. The similar phenomena of Ag diffusion in SiO_2_ layer by *in situ* TEM observation have been reported by Yang et al. [34]. The Cu diffusion in ZrO_2_ layer by TEM observation was also reported by other group [35]. The number of successful devices with different device sizes ranging from 0.4 × 0.4 to 8 × 8 μm^2^ is shown in Table 1. The device size of less than 2 μm can carry current of 10 mA, while the larger size of 4-μm device can carry high current of 70 mA. Most important thing is that the larger size devices show 100% success, while the failure is increased with decreasing device size. It is expected that stronger Cu pillar is needed for 3D integration of cross-point nonvolatile memory. This will be easy way and low cost for application of 3D cross-point memory [32]. Therefore, we need those devices which can sustain at high current for long time, and we find that, the devices with large area are compatible for this purpose. Figure 3b shows the statistical distribution of currents at low resistance state (LRS) for the device-to-devices. The mean value  and the standard deviation (*σ*) of currents for the 4-μm devices at a read voltage (*V*_read_) of 1 V are 49.96 and 9.33 mA, while those values for the 8-μm devices are 46.14 and 6.61 mA, respectively. The read current of the 8-μm devices is slightly lower than that of the 4-μm devices owing to lower formation voltage. This implies that small amount of Cu diffusion into the Al_2_O_3_ films for the larger size devices than the smaller sizes. However, uniformity of the high current carrying Cu pillars is better for the 8-μm devices than those of the 4-μm devices. The mechanism of Cu pillar formation inside the pristine device is as follows. These are basically the CBRAM devices; however, 20-nm-thick Al_2_O_3_ film is studied for demonstration, and further study for real application of the Cu pillars into the 1-μm-thick Al_2_O_3_ films is necessary. When the positive bias is applied on the active Cu electrode, the Cu^z+^ (*z* = 1,2) ion is formed by oxidation, then those ions migrate through the switching medium in the presence of high electric field, and finally, they become reduced at the TiN BE. This formation process transforms the pristine device from its initial resistance state (IRS) to LRS as well as stronger Cu pillar is formed. By applying negative voltage on the TE, the Cu pillars of some smaller size devices are dissolved because of Joule heating. Robust Cu pillars have been investigated by measuring endurance properties below.

**Table 1:** **Device size-dependent success and failure of the Cu pillars**

Device size (μm ^2^)	Current compliance (mA)	Number of successful devices (%)
0.4 × 0.4	10	74
1 × 1	10	78
2 × 2	10	98
4 × 4	70	100
8 × 8	70	100

**Figure 4:**
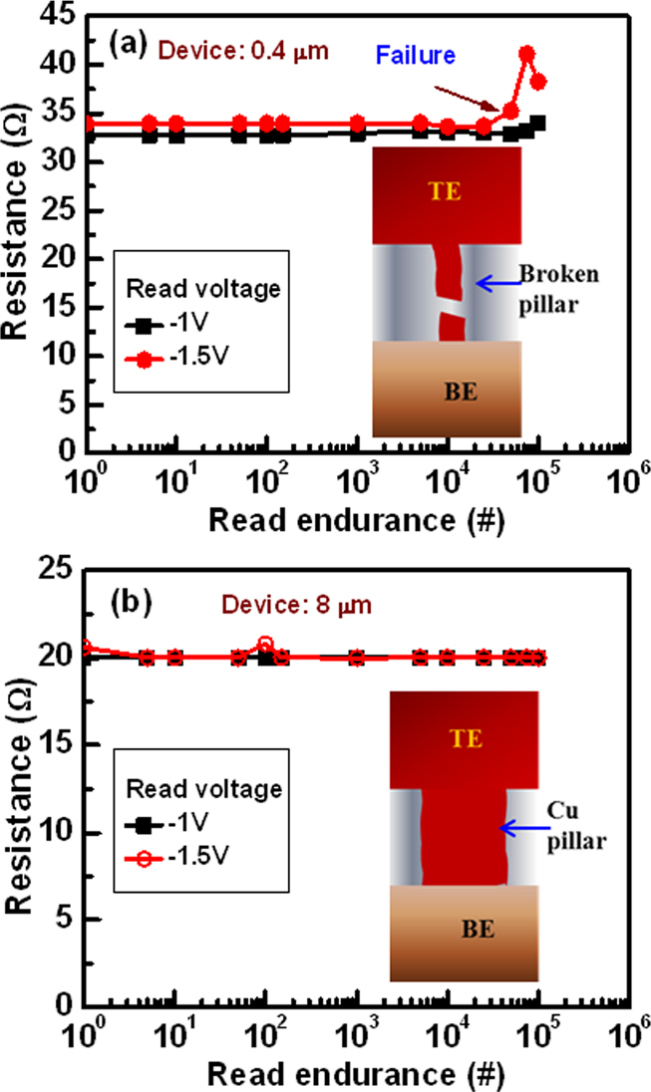
**Read pulse endurance characteristics. (a)** Read pulse endurance properties degraded at high negative voltage due to the Joule heating phenomena for the smallest size devices. The Cu pillar is broken during read endurance test, which is shown in schematic view. **(b)** For the large size devices, long endurance reveals the robustness of the Cu pillars inside the switching medium at a *V*_read_ of -1.5 V. Long read endurance of 10^5^ cycles is obtained for the 8-μm devices. A stronger Cu pillar is formed into the Al_2_O_3_ films, which is shown in schematic view inside of figure.

Figure 4 shows the read endurance characteristics with different negative read voltages. As it is bipolar device, the negative bias makes the RESET. After formation, we have increased the negative bias sequentially as -1 and -1.5 V on the TE. The current compliances are 10 and 70 mA for the 0.4- and 8-μm devices, respectively. For the 0.4-μm devices, a value of LRS is approximately 32 Ω (Figure 4a), while the value is approximately 20 Ω for the 8-μm devices (Figure 4b). This indicates that the diameter of Cu pillar is larger for the 8-μm devices than the 0.4-μm devices, as shown schematic view in the inset. For the 0.4-μm devices, the LRS state is increased after approximately 40 and 30 k cycles for the read voltages of -1 and -1.5 V, respectively. The Cu pillar is broken easily after higher negative voltage on the TE, as shown schematically in the inset of Figure 4a. Robust read pulse endurances of >10^5^ cycles are obtained for the 8-μm devices because larger diameter of the Cu pillars, as shown schematically in the inset of Figure 4b. So, after formation of the conducting path, the possibility of deterioration of the paths is less which indicates the ability of Cu pillar for 3D cross-point architecture in the future. Beside the Cu pillar investigation, the resistive switching characteristics of the Cu/Al_2_O_3_/TiN CBRAM devices with smaller thickness (<10 nm) of Al_2_O_3_ layer are also important, which have been investigated below.

**Figure 5:**
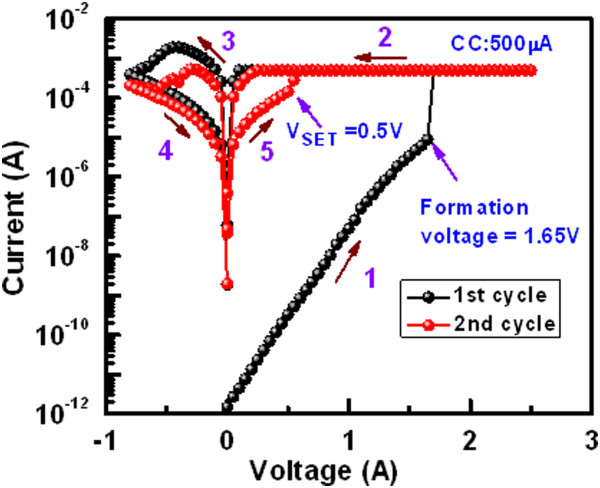
***I*** **-** ***V*** **switching characteristics.** Typical current-voltage characteristics of the 8-μm devices with a 2-nm-thick Al_2_O_3_ film at a CC of 500 μA. A low formation voltage of 1.65 V is observed.

**Figure 6:**
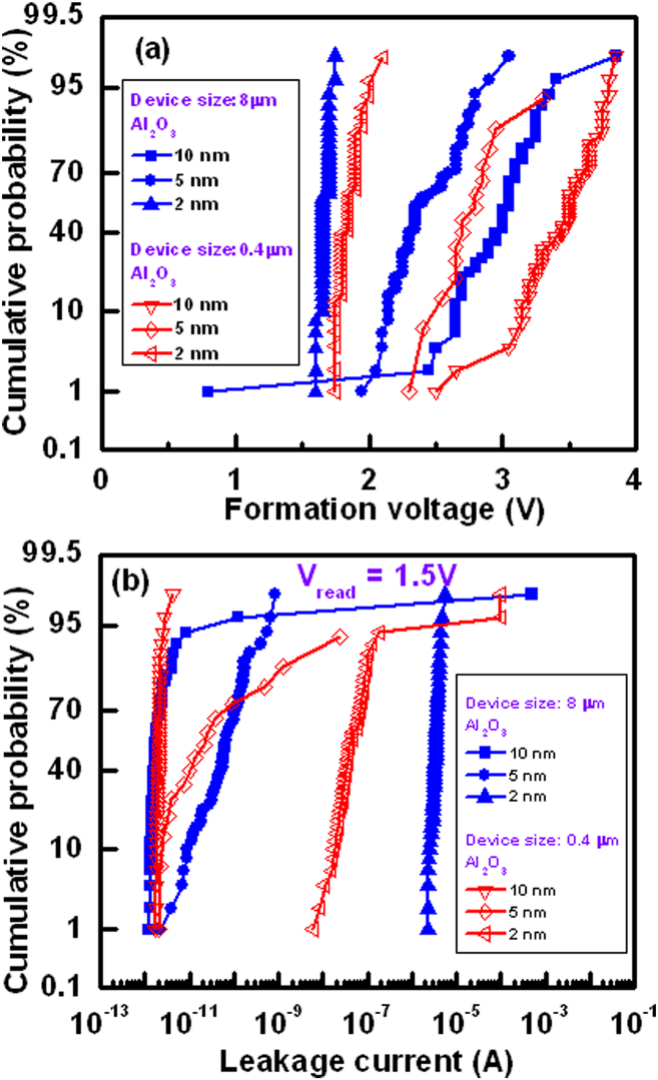
**Cumulative probability of formation voltage and leakage current. (a)** Formation voltages and **(b)** leakage currents depend on the device sizes and thickness of the Al_2_O_3_ films for the Al/Cu/Al_2_O_3_/TiN CBRAM devices with sizes of 8 × 8 and 0.4 × 0.4 μm^2^. The thicknesses of Al_2_O_3_ film are 2, 5, and 10 nm.

Figure 5 shows the typical *I*-*V* curves of the 8-μm devices with a 2-nm-thick Al_2_O_3_ film and a CC of 500 μA is applied. The sweeping voltage is shown by arrows 1 to 5. A low *V*_form_ of 1.65 V is observed for this thin Al_2_O_3_ film. Cumulative probability of 50 CBRAM devices with different size and thickness of the Al_2_O_3_ films is plotted (Figure 6a). The average values of *V*_form_ are 1.7, 2.4, and 3 V for the 8-μm devices while those values are 1.85, 2.7, and 3.4 V for the 0.4-μm devices with different thicknesses of Al_2_O_3_ film of 2, 5, and 10 nm, respectively. Those values of *V*_form_ are lower than the 20-nm-thick Al_2_O_3_ films (Figure 3a). For the 2-nm-thick Al_2_O_3_ films, tight distribution of *V*_form_ is found to be 1.6 to 1.75 V and 1.75 to 2.1 V for the 8- and 0.4-μm devices, respectively. Figure 6b shows cumulative probability of the leakage currents for the 8- and 0.4-μm devices with thicknesses of the Al_2_O_3_ films of 2, 5, and 10 nm. The leakage currents at 50% probability are found to be 3.4 μA, 60 pA, and 1.7 pA for the 8-μm devices while those values are found to be 39 nA, 22 pA, and 2.1 pA for the 0.4-μm devices with thicknesses of the Al_2_O_3_ films of 2, 5, and 10 nm, respectively. The 10-nm-thick Al_2_O_3_ films show device size-independent leakage currents, which is due to the limit of current measurement by our probe station. It is found that the variation of formation voltage is directly proportional to the switching material thickness and inversely proportional to the device size area. On the other hand, the leakage current shows the opposite nature of the formation voltage. It varies directly proportional to the device size and inversely proportional to the switching materials' thickness. It happens because the reduction in device size causes the decrement of defects inside the switching material which in turns increases its insulation property. This causes the leakage current lower, and so, the required voltage to change its resistance state is more. The reduction in switching material thickness causes the higher possibility of electron tunneling through the insulator layer which causes the enhancement in leakage current. It is observed that the 2-nm-thick Al_2_O_3_ films show better uniformity of the formation voltages as well as the leakage currents. Both the RESET current (*I*_RESET_) and voltage (*V*_RESET_) at first cycle are found to be approximately 2 mA and -0.45 V, respectively (Figure 5). The SET voltages *V*_SET_, *V*_RESET_, and *I*_RESET_ at the second cycle are lower 0.5 V, -0.3 V, and approximately 540 μA than those of the values that are observed in the first cycle, respectively. The *I*_RESET_ is slightly higher than the current compliance because of thinner (2 nm) Al_2_O_3_ film. To dissolve more length of the Cu filaments or to increase high resistance state (HRS), higher negative voltage of -0.8 V is required. *I*-*V* curves imply that the RESET is happened through a slow deterioration process of the existing metallic filaments in its weak point by reduction due to the negative bias on the TE. A resistance ratio (HRS/LRS) at a *V*_read_ of 0.1 V is found to be 16, which is acceptable for application.

**Figure 7:**
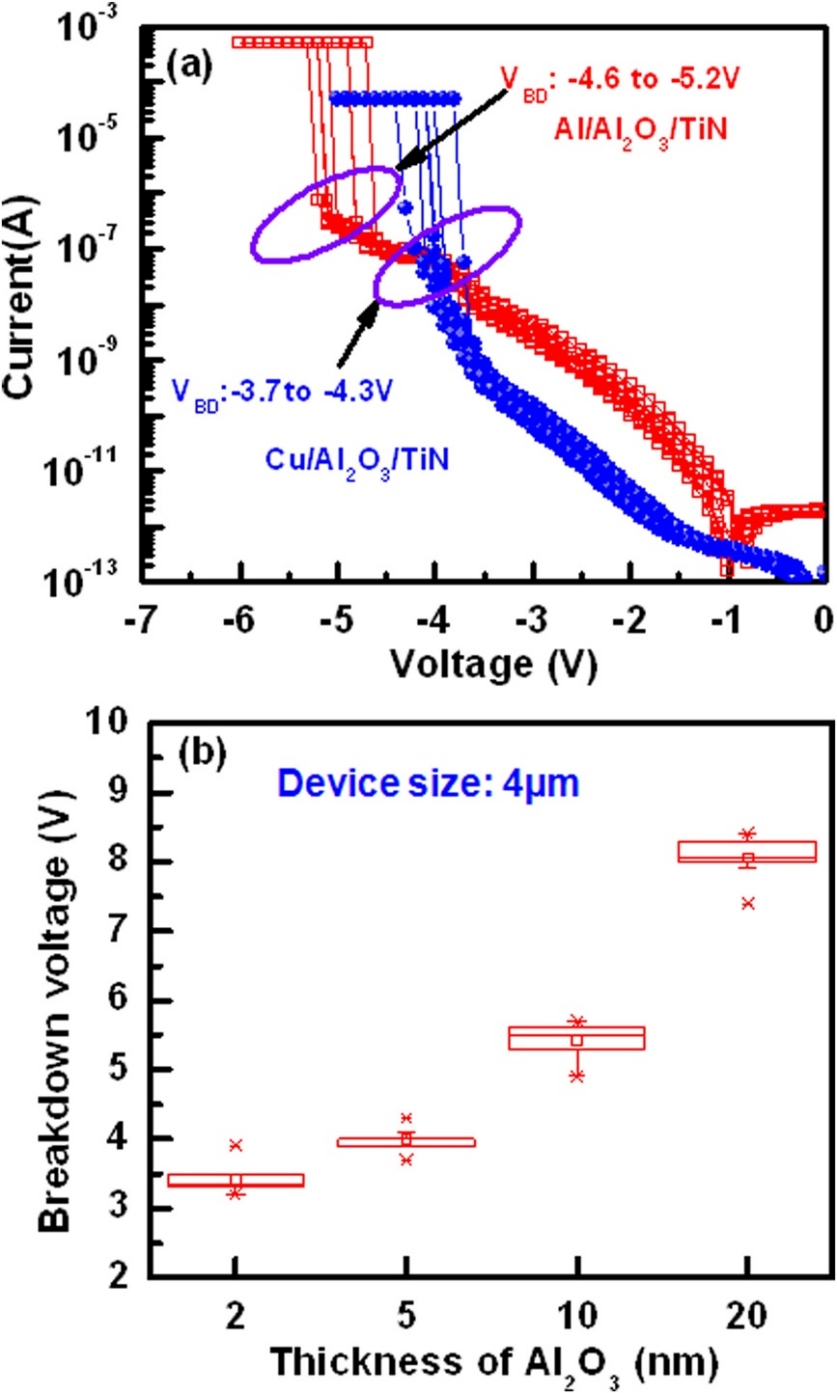
**Thickness-dependent Al**_**2**_ **O**_**3**_ **film breakdown phenomena with Cu and Al top electrodes. (a)** *I*-*V* characteristics show the breakdown voltage of the Cu/Al_2_O_3_/TiN and Al/Al_2_O_3_/TiN structures. The higher breakdown voltage of Al/Al_2_O_3_/TiN than that of the Cu/Al_2_O_3_/TiN structure is owing to oxidized Al at the Al/Al_2_O_3_ interface during deposition by thermal evaporator. **(b)** The breakdown voltage of the Al/Cu/Al_2_O_3_/TiN structures increases with increasing the thickness of Al_2_O_3_ film.

As we have mentioned, the switching mechanism is based on Cu filament formation/dissolution into the Al_2_O_3_ film under external bias, and this could be also understood indirectly by studying the breakdown phenomenon using two different top electrodes viz. Cu and Al, as shown in Figure 7. The thickness of the Al_2_O_3_ films is 5 nm. The average breakdown voltage (*V*_BD_) of the randomly measured ten devices of the Al/Al_2_O_3_/TiN structures is -4.99 V (i.e., -4.6 to -5.2 V) whereas this value of the Al/Cu/Al_2_O_3_/TiN structures is 3.99 V (i.e., -3.7 to -4.3 V), as shown in Figure 7a. The value of *V*_BD_ is higher for the Al TE because the Al makes an additional oxide layer at the Al/Al_2_O_3_ interface. According to our previous report [36], the AlO_*x*_ layer was formed at the Al/TaO_*x*_ interface. It is also found that the value of *V*_BD_ for the Al/Cu/Al_2_O_3_/TiN structures increases with increasing the thickness of Al_2_O_3_ layer, as shown in Figure 7b. If one can compare between the breakdown voltage and the formation voltage of the Al/Cu/Al_2_O_3_/TiN structures with a 2-nm-thick Al_2_O_3_ layer, then the average value of breakdown voltage is higher than the formation voltage (-3.2 vs. 1.85 V). The similar trend is observed for all thicknesses of the Al_2_O_3_ films, as discussed above. This result reveals that the formation takes place due to the Cu ion migration through the Al_2_O_3_ layer. Under high electric field approximately 10^7^ V/cm before breaking the stable Al-O bonds, electrochemically active Cu ions diffuse easily through the Al_2_O_3_ layer and make a metallic path under a low positive voltage applied on the TE. The Cu ion migration as well as filament formation into different switching layers under external bias was also reported by other groups [16–18, 24]. However, the switching uniformity is important of these CBRAM devices, which have been explained below.

**Figure 8:**
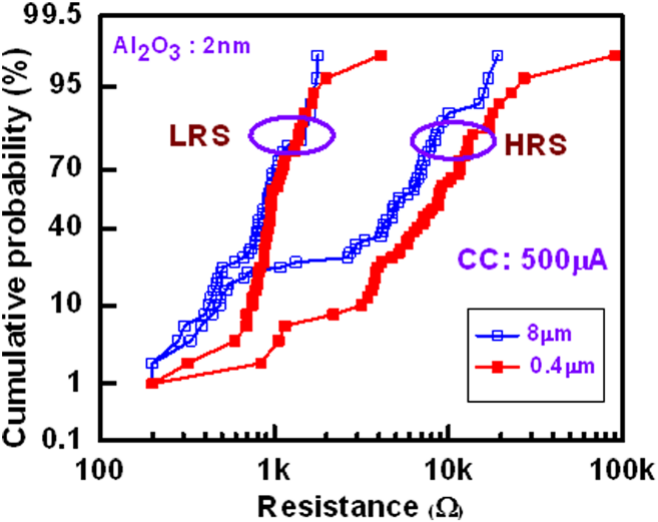
**Cumulative probability of HRS and LRS.** The smaller size device shows superior uniformity than that of the larger size devices. It is observed that the 0.4-μm devices show 88% success for switching. The data read on the second switching cycle.

**Figure 9:**
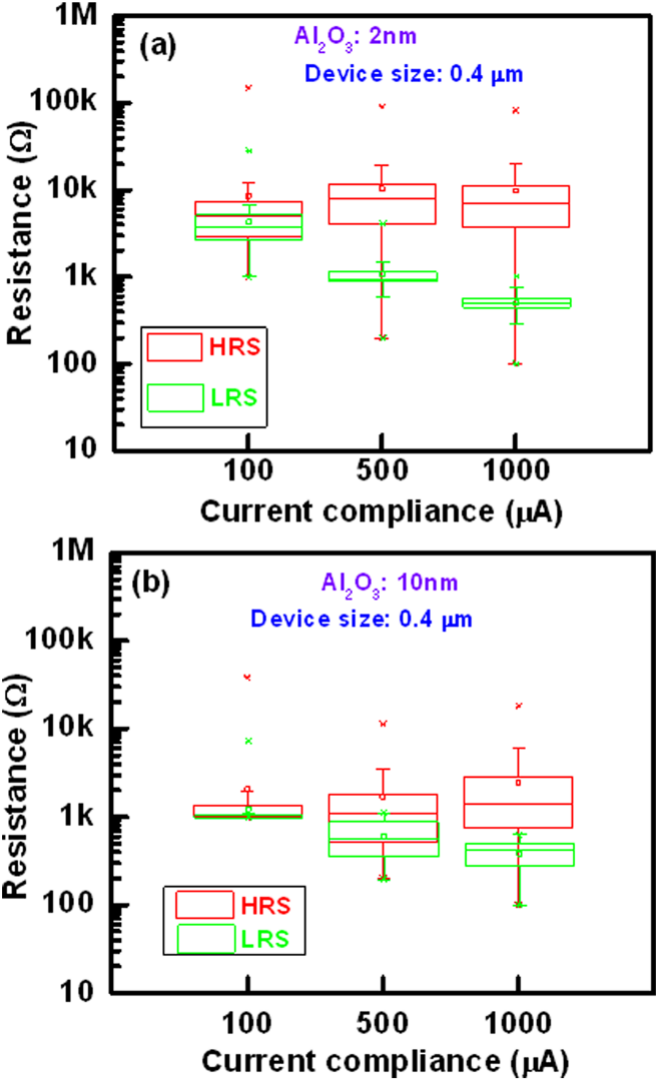
**Statistical distribution of HRS and LRS.** The thicknesses of Al_2_O_3_ film are **(a)** 2 nm and **(b)** 10 nm. The LRS decreases with increasing the CCs of 100 to 1,000 μA for the 0.4-μm devices. The 2-nm-thick Al_2_O_3_ film shows superior resistance ratio than that of the 10-nm-thick films.

**Figure 10:**
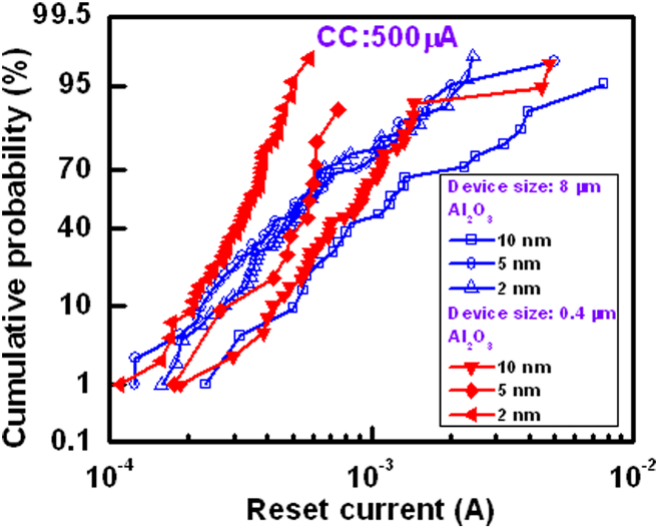
**RESET current distribution.** The RESET currents depend on the thickness of the Al_2_O_3_ films and device sizes. The 0.4-μm devices with a 2-nm-thick Al_2_O_3_ film show the lowest RESET current distribution as compared to the others at a CC of 500 μA.

Figure 8 shows cumulative probability of device-to-devices. The HRS and LRS for the 8- and 0.4-μm devices with a 2-nm-thick Al_2_O_3_ film are plotted. The average values of  at HRS and LRS are 5.34/4.44 kΩ and 895/407 Ω for the 8-μm devices, respectively, while those values are 10.3/12.9 kΩ and 1.07/539 kΩ for the 0.4-μm devices, respectively. The value of LRS is slightly lower for the 8-μm devices than the 0.4-μm devices, which is owing to higher diffusion rate of Cu ion into the Al_2_O_3_ film under external bias. By considering the resistance ratio of >2, the 0.4-μm devices show higher switching yield than that of the 8-μm devices (88% vs. 74%). This suggests that the 0.4-μm devices have good switching uniformity. Figure 9 shows the statistical distribution of resistance states with different current compliances of 100, 500, and 1,000 μA for the 2- and 10-nm-thick Al_2_O_3_ films. Except few devices or without proper sweeping voltage/current, there is no memory window at a CC of 100 μA. However, the value of LRS decreases and HRS remains almost the same with increasing the CCs (Figure 9a,b). The resistance ratio increases with increasing the CCs. Table 2 represents the average values of LRS, HRS, and HRS/LRS for the 8- and 0.4-μm devices with different thicknesses of Al_2_O_3_ film of 2, 5, and 10 nm. To obtain the average values, 50 CBRAM devices were measured. It is obvious that the resistance ratio is higher at CC of 1 mA as compared to the value at a CC of 500 μA because of lower LRS value. At a CC of 500 μA, a high resistance ratio of 9.6 is obtained for the 0.4-μm devices with a 2-nm-thick Al_2_O_3_ film. In this case, more switchable devices are obtained (Figure 8), which is due to better control of Cu migration under external bias. The values of LRS are decreased with increasing both the device size and thickness of the Al_2_O_3_ films at a CC of 500 μA (Table 2), which can be explained by *I*_RESET_ later. Figure 10 shows cumulative probability of the RESET currents for the 8- and 0.4-μm devices with thicknesses of the Al_2_O_3_ films of 2, 5, and 10 nm at a CC of 500 μA. The average *I*_RESET_ values of the 2-, 5-, and 10-nm-thick Al_2_O_3_ films are found to be 706.1, 749.4, and 1,690 μA, respectively, for the 8-μm devices, while those values are found to be 327.5, 505.4, and 1,020 μA, respectively, for the 0.4-μm devices. It is observed that the *I*_RESET_ value decreases with decreasing the thickness of the Al_2_O_3_ films. Considering the thickness-dependent formation voltage (Figure 6a), the Cu ion can migrate more in the thicker Al_2_O_3_ films, resulting larger diameter of Cu filament. That is why the thicker Al_2_O_3_ film has higher RESET current. A lowest average RESET current of 327.5 μA with good uniformity is obtained for the 0.4-μm devices with a 2-nm-thick Al_2_O_3_ film (Figure 10). As mentioned above, the formation voltage of the thinner Al_2_O_3_ films is lower than that of the thicker one. Under SET, small amount of Cu will be migrated for the thinner Al_2_O_3_ films as well as thinner diameter of the Cu filaments. That is why the LRS value of the thinner Al_2_O_3_ films is larger than the thicker one. Under RESET, the total length of the Cu filaments will be dissolved for the thinner Al_2_O_3_ films because of both higher electric field and thinner filament diameter than that of the thicker one. On the other hand, interface-type switching or even no RESET is observed for the thicker Al_2_O_3_ films. Therefore, HRS value of the thinner Al_2_O_3_ films is higher than those of the thicker one. It can be concluded that thicker Al_2_O_3_ film can be used for the Cu pillars to apply in 3D cross-point memory and thinner one can be used for the nonvolatile resistive switching memory, and data retention test is shown below.

**Table 2:** **Device size- and thickness-dependent LRS, HRS, and resistance ratio with different current compliances**

Thickness of Al _2_O _3_(nm)	Device size (μm ^2^)	Average value (Ω) and resistance ratio of HRS/LRS					
		CC: 500 μA			CC: 1 mA		
		LRS	HRS (k)	HRS/LRS	LRS	HRS (k)	HRS/LRS
2	0.4 × 0.4	1070	10.3	9.63	502	9.8	19.55
	8 × 8	895	5.34	5.97	384	22.3	58.10
5	0.4 × 0.4	826	4.64	5.62	487	3.6	7.39
	8 × 8	652	4.54	6.96	364	18.3	50.27
10	0.4 × 0.4	572	1.71	3.00	394	2.48	6.3
	8 × 8	492	1.48	3.00	343	2.05	6.0

**Figure 11:**
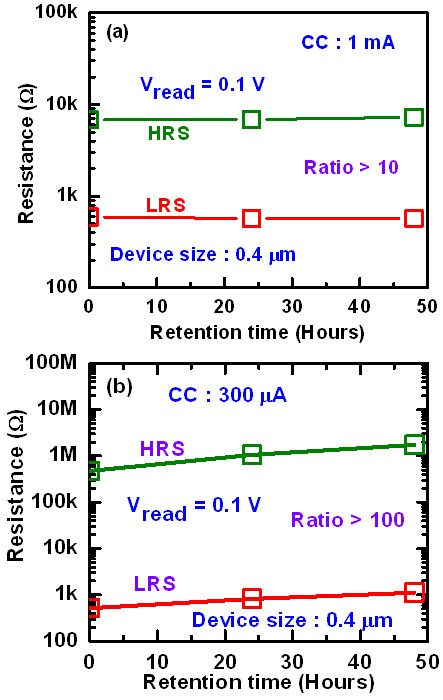
**Data retention characteristics.** Good data retention of >48 h is obtained for the CBRAM devices at CC of **(a)** 1 mA and **(b)** 300 μA. The thickness of the Al_2_O_3_ layer is 2 nm.

**Figure 12:**
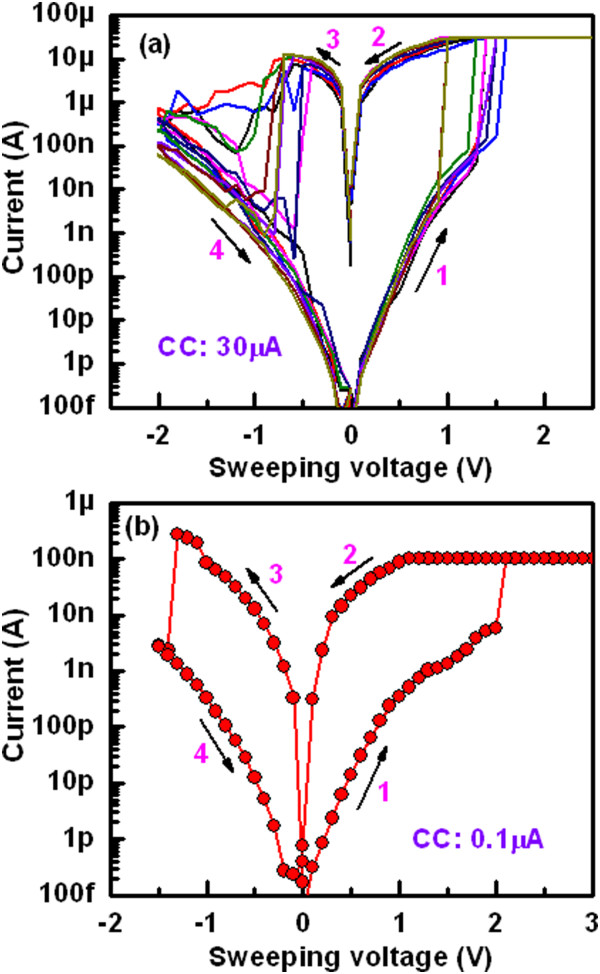
***I*** **-** ***V*** **curves a low CC of 30 μA.** Typical *I*-*V* characteristics under a CC of **(a)** 30 μA with a thickness of the Al_2_O_3_ layer of 5 nm and **(b)** at a lowest CC of 0.1 μA. Forming-free *I*-*V* characteristics are observed. A high resistance ratio of >10^4^ at a read voltage of 0.2 V is also obtained. The voltage sweeping direction is shown by arrows 1 to 4.

Figure 11 shows data retention characteristics for the 0.4-μm devices with a 2-nm-thick Al_2_O_3_ film. It is found the stable retention characteristics of >48 h at a CC of 1 mA (Figure 11a). The LRS is increased (slightly) with retention time, however, long time retention of 48 h at a CC of 300 μA is obtained (Figure 11b), which may be the higher dissolution rate of the existing filament at lower CC. The resistance ratio for a CC of 300 μA is higher than the ratio at CC of 1 mA (100 vs. 10). At a lower CC, the small amount of the Cu atoms is responsible for the conducting filament formation. If small amount of Cu atoms from the thinner filament may be dissolved by neighbor defects into the AlO_*x*_ film or dissolved by reading data, then both HRS and LRS could be increased with time. For larger diameter of the Cu filaments under higher CC, it shows stable with time because dissolution of small amount Cu from the filaments does not affect the filament resistance of LRS, or even HRS. However, further study is needed to form a stronger Cu filament with thinner diameter. By adjusting measurement parameters, this CBRAM device shows forming-free *I*-*V* characteristics under a low CC of 30 μA and a RESET current of <30 μA with a high resistance ratio of >10^5^ at a read voltage of +0.2 V (Figure 12a). This device is operated even at a lowest CC of 0.1 μA (Figure 12b) with a large resistance ratio of >10^4^, which is very useful for future nanoscale nonvolatile memory applications.

## Conclusions

The device size- and thickness-dependent Cu pillars and resistive switching memory characteristics using the Al/Cu/Al_2_O_3_/TiN CBRAM devices have been investigated. The stronger Cu pillars with yield of 100% are formed into the Al_2_O_3_ films for the larger size devices at a high CC of 70 mA, which is due to the easy heat dissipation effect. Robust Cu pillar with a long endurance of >10^5^ cycles is obtained even a high negative voltage of -1.5 V, which promises for future 3D cross-point memory applications. Improved resistive switching memory characteristics such as high switching yield of 88%, low voltage operation of ±2 V, and lower average RESET current of 327.5 μA for a CC of 500 μA are obtained for the 0.4-μm devices with a 2-nm-thick Al_2_O_3_ film. The formation/dissolution of the Cu filaments into a 2-nm-thick Al_2_O_3_ film is responsible to have better switching characteristics under external bias because of thinner film. On the other hand, Cu diffusion rate is higher for the larger size and thicker film which will have inferior switching characteristics, however, give us superior Cu pillars. Good data retention characteristics of 48 h are obtained at a CC of 300 μA. This device shows forming-free *I*-*V* characteristics under a lowest CC of 0.1 μA with a high resistance ratio of >10^4^. This strategy on the Cu pillars and resistive switching memory characteristics of the Cu/Al_2_O_3_/TiN CBRAM devices will help to develop in future 3D cross-point architecture with low cost applications.
